# Supplementary material for: Global hypo-methylation in a proportion of glioblastoma enriched for an astrocytic signature is associated with increased invasion and altered immune landscape
Source: eLife. 2022 Nov 22;11:e77335. doi: 10.7554/eLife.77335 (PMC9681209; doi:10.7554/eLife.77335)
Supplement: Figure 2—source data 1. [file elife-77335-fig2-data1.zip › Figure_2_source_data_1/Figure_2A/homerResults/motif47.similar.html]

motif47

## Information for motif47

A
C
G
T
A
G
T
C
G
T
A
C
A
C
G
T
A
G
T
C
C
G
T
A
C
G
A
T
A
G
T
C
  
Reverse Opposite:  

A
C
T
G
C
G
T
A
A
C
G
T
A
C
T
G
C
G
T
A
A
C
T
G
A
C
T
G
C
G
T
A
  

|  |  |
| --- | --- |
| p-value: | 1e-13 |
| log p-value: | -3.057e+01 |
| Information Content per bp: | 1.918 |
| Number of Target Sequences with motif | 87.0 |
| Percentage of Target Sequences with motif | 3.75% |
| Number of Background Sequences with motif | 55.1 |
| Percentage of Background Sequences with motif | 1.51% |
| Average Position of motif in Targets | 106.1 +/- 52.1bp |
| Average Position of motif in Background | 108.9 +/- 54.1bp |
| Strand Bias (log2 ratio + to - strand density) | -0.4 |
| Multiplicity (# of sites on avg that occur together) | 1.06 |
| Motif File: | file (matrix) reverse opposite |

### Similar de novo motifs found

|  |  |  |  |  |  |  |  |
| --- | --- | --- | --- | --- | --- | --- | --- |
| Rank | Match Score | Redundant Motif | P-value | log P-value | % of Targets | % of Background | Motif file |
| 1 | 0.649 | T G C A T C G A G T C A C G A T T C A G C T G A C T A G T C A G C G T A C T G A C T G A T A G C | 1e-12 | -28.081393 | 0.69% | 0.06% | motif file (matrix) |
| 2 | 0.654 | A C T G A C G T A C T G C G T A C G T A A C T G C G T A A C T G A C T G C G T A C G T A A C T G | 1e-10 | -23.057069 | 0.60% | 0.07% | motif file (matrix) |
